# Three-Dimensional Refractivity Model for Atmospheric Mitigation in Distance and Vertical Angle Measurements

**DOI:** 10.3390/s25071981

**Published:** 2025-03-22

**Authors:** Raquel Luján, Luis García-Asenjo, Sergio Baselga

**Affiliations:** Department of Cartographic Engineering, Geodesy and Photogrammetry, Universitat Politècnica de València, Camino de Vera s/n, 46022 Valencia, Spain; ralugar@cgf.upv.es (R.L.); lugarcia@cgf.upv.es (L.G.-A.)

**Keywords:** atmospheric refraction, geodetic measurements, TS, meteorological sensors, ERA5

## Abstract

Atmospheric refraction is a significant challenge to accurate distance and angle measurements in open-air environments, often limiting the precision of measurements obtained using electro-optic geodetic instruments despite their nominal accuracies. This study introduces a novel model, 3D-RM, designed to mitigate atmospheric effects on both distance and vertical angle measurements. The 3D-RM integrates in situ meteorological data from a network of automatic data-loggers, terrain information from a digital terrain model (DTM), and sensible heat flux from the fifth generation of European Centre for Medium-Range Weather Forecast reanalysis (ERA5), which is used in the application of the Turbulence Transfer Model (TTM) for estimating vertical refractivity gradients at various height levels. The model was tested with total station observations to 10 target points during two field campaigns. The results show that applying the model for distance correction leads to improvements in terms of closeness to reference values when compared to the standard method, which relies only on meteorological data collected at the station. Furthermore, the model has been additionally tested by removing the station meteorological data (3D-RM2). The results demonstrate that accurate corrections can be obtained even without the need of meteorological sensors specifically installed at the station point, which makes it more flexible. The 3D-RM is a cost-effective and relatively easy-to-implement solution, offering a promising alternative to existing methodologies, such as measuring meteorological values at both station and target points or the development of new instruments that can compensate the refractivity (such as a multiple-color electronic distance meter).

## 1. Introduction

Nowadays, fields like industrial and dimensional metrology benefit from instruments that can provide submillimetric measurements under well-controlled indoor conditions. However, challenges arise when accurate open-air measurements over distances from a few hundred meters to several kilometers are required. This is the case of critical applications like deformation monitoring by using high-precision surveying techniques such as electronic distance measurements (EDMs) and total station measurements (TS). Regardless of the technique, atmospheric refraction can often be a limiting factor in achieving the nominal accuracy of the instrument [1].

The traditional approach to mitigate atmospheric refraction on EDMs involves estimating the index of refraction from empirical equations [2,3,4,5] based on meteorological parameters at both ends of the baseline. Then, the average of their resulting refraction indexes is used to compute the correction. However, this approach has proven insufficient due to the inadequate representation of the atmospheric conditions along the entire path [6,7]. These limitations are especially pronounced in complex terrains where the atmospheric conditions can vary significantly along the path. Furthermore, target points are frequently inaccessible, thus impeding the installation of meteorological sensors. Similarly, operational limitations can make difficult the use of sensors in all stations.

To overcome this problem, new instruments and methodologies have been proposed, including the development of two-wavelength distance meters [8,9] or the use of a GNSS (Global Navigation Satellite System) as a distance meter (GBDM+) [10,11]. While these innovative approaches are still under development, the need to improve atmospheric corrections in terrestrial geodetic techniques (EDMs, TS) persists.

The difficulty of properly modeling the atmospheric effects in distances and angles lies in the complexity of the physical processes, especially in the boundary layer, which is the lowest part of the atmosphere, where there is significant interaction between the ground and the atmosphere. Extensive studies on these processes have been conducted since the 1950s [12,13,14]. Particularly during the 1980s, the Turbulence Transfer Model (TTM) was proposed as a basis for refraction mitigation in several geodetic techniques [15,16,17]. Despite these early foundations, atmospheric refraction remains a significant challenge for geodetic applications. Recent studies have analyzed the impact of refraction in new technologies such as terrestrial laser scanners (TLSs) [18,19] and high-precision total station measurements [20]. Refraction in dam deformation monitoring is also a field of interest [21]. Additionally, efforts to refine vertical angle correction methodologies [22] reflect the ongoing need to improve atmospheric models across different geodetic techniques.

Leveraging this extensive research, the present study applies the TTM as presented in [23], which provides equations for deriving refractivity gradients from parameters measured in the field (i.e., air temperature, pressure, humidity, heat flux, and wind speed). While the theoretical basis of the TTM has been long established, recent technological advances have created new opportunities for this practical application. The development of atmospheric sensors capable of automatic data recording at a relatively low cost, coupled with the availability of new global remote sensing products, and increased computational power make it possible to revisit and refine classical concepts like TTM for its integration into new refractivity correction models suitable for automatic processing.

In this paper, we introduce a 3D refractivity model, from now on called 3D-RM, based on the use of a network of meteorological sensors, ERA-5 sensible heat flux data, terrain information from a Digital Terrain Model (DTM), and the application of a simple approximation of the Turbulence Transfer Model. The proposed 3D model allows for the estimation of the refractivity and the refractivity gradient at the desired points within the working area. Those values are subsequently integrated to automatically obtain corrections for both distance and vertical angle measurements. The model is designed as a cost-effective solution for situations where installing meteorological sensors at critical points is not feasible or when such installations do not adequately represent the conditions along the line of sight. This addresses current limitations in refractivity modeling that hinder high-precision surveying techniques from reaching optimal accuracy.

The 3D-RM has been tested in two field experiments in the area of La Muela de Cortes de Pallás, Spain. These experiments use total station measurements (TS) and targets located at 10 control points (CPs) whose coordinates are known from previous campaigns.

This paper is organized as follows. Section 2 presents the theoretical background, including a brief review of atmospheric refraction and ways to mitigate it, the main aspects of the Turbulence Transfer Model, and sensible heat flux information. Section 3 introduces the newly proposed refractivity model. Section 4 describes the experimental validation of the model. The results are shown in Section 5 and, finally, some conclusions and future work are summarized in Section 6.

## 2. Theoretical Background

### 2.1. Refraction Effects in Geodetic Measurements

Atmospheric refraction, which is the phenomenon through which optical signals traveling through the atmosphere are affected by the inhomogeneity of the air, plays a significant role in geodetic measurements, resulting in deviations in both distance and angle measurements.

This problem has been extensively discussed over decades, with significant research during the 1980s (e.g., [17]).

One aspect of atmospheric influences on electromagnetic waves is the variability in the speed of propagation due to variations in the index of refraction (n) along the line of sight (LoS). This directly affects distance measurements made by geodetic instruments (e.g., EDMs). In practice, refractivity (N) is often used instead of the index of refraction (n), being N = 10^6^ (n − 1). The refraction variability often induces distance variations of tens of parts per million (ppm), that is, several cm in a distance of one km. Therefore, an accurate determination of the refractive index or refractivity along the line of sight is essential for correcting distance measurements.

Changes in the index of refraction are directly related to variations in meteorological parameters, mainly temperature and pressure, which have the biggest effect on visible light and near-infrared waves. Humidity plays a smaller role and CO_2_ concentration typically has a negligible impact. However, they have to be considered in special applications requiring accuracies of 10^−7^ [3,4].

The influence of meteorological parameters on measured distances has been a subject of study since the development of EDM technology, yielding different correction models. More recently, studies such as that of Sabzali and Jazirian [24] have revisited this topic, analyzing the impact of temperature, pressure, and humidity on measured distances and reviewing different correction models, emphasizing the importance of temperature and highlighting the limitations of using only meteorological parameters at the baseline ends. Rodriguez et al. [25] presented a real-time temperature acquisition system for distance correction. It was tested on monitoring distances of a 2D network. The results showed significant temperature variation within the working area and demonstrated improved accuracy when combining all the temperature data instead of using one single value.

Changes in the atmospheric parameters are also influenced by the topography of the area. Artese and Perrelli [20] developed a model using a Digital Terrain Model (DTM) and historical climate data to characterize daily and seasonal oscillations in the area and then correct measurements, resulting in significant improvements in distance standard deviations.

Aside from speed variations, refraction also introduces errors in measured angles due to the bending of rays. Variations in angles are related to the refractivity gradients, both vertical and horizontal. While vertical refraction has been the object of numerous studies, horizontal refraction is usually neglected in open-air geodetic works owing to its normally lower impact, even though there are specific contexts, like tunneling, where horizontal refractivity gradients can be significant [26].

Vertical refraction can be also related to meteorological parameters. Gaifillia et al. [27] and Baselga et al. [28] derived empirical models to estimate the value of the refraction coefficient based on meteorological data. Nikolitsas and Lambrou [22] presented a comparison between two different methodologies for correcting vertical angles, one focused on the measurement of temperature gradients for determining the refraction coefficient, and the other based on trigonometrical leveling network adjustment. The study highlighted the challenges in measuring the temperature gradients.

Hirt et al. [29] conducted a study based on simultaneous reciprocal vertical angle measurements, an approach known for over a century (e.g., [30]). While the methodology is well established, their work demonstrated the significant variability in the coefficient of refraction in different local conditions, underlining the importance of addressing site-specific atmospheric effects for improved accuracy.

The idea of modeling atmospheric refraction using different vertical layers with distinct properties is not new. Angus-Leppan [31] empirically established values for the coefficient of refraction at various heights. More recently, Kerekes [29] studied the effects of temperature, pressure, and temperature gradient on Terrestrial Laser Scanner (TLS) measurements, using a layer-based approach to describe the vertical variability in these parameters.

The contribution of this work is the use of all this well-established theory to implement a model capable of providing distance and vertical angle corrections. This model characterizes the local low part of the atmosphere by using in situ meteorological measurements along with sensible heat flux from ERA5 and supplementary terrain information from a digital terrain model (DTM).

### 2.2. Turbulence Transfer Model (TTM)

The Turbulence Transfer Model (TTM) presented by Dodson and Zaher [23] and, in turn, based on a prior model by Priestley [14] is a model of the structure of atmospheric temperature taking into account the analysis of heat fluxes transfer. This model provides equations for calculating potential temperature gradients within various atmospheric regions, which are defined based on certain in situ parameters.

The calculation of the potential temperature (θ) depends on the stability of the atmosphere. A straightforward stability criterion is the value of the sensible heat flux (*H*, in W/m^2^): the atmosphere is neutral when *H* is 0, stable when *H* is negative, and unstable when *H* is positive [32].

Positive values of sensible heat flux indicate a transfer from the ground to the air, which commonly happens during the daytime when the surface is heated by the sun. Conversely, at night, when the ground cools and the heat transfer is directed from the air to the surface, heat flux presents negative values, associated with a stable atmosphere.

In a neutral atmosphere, the potential temperature gradient (*∂θ/∂h*) is 0. However, in stable and unstable atmospheres, specific formulas are employed. All equations provided in this subsection follow the formulations described by Dodson and Zaher [23].

- Stable atmosphere

(1) $$\frac{\partial \theta }{\partial h}=-2\cdot 10^{-3}\cdot \frac{H}{U_{*}\cdot h}\cdot \left(1+\frac{5\cdot h}{L}\right)$$

- Unstable atmosphere

(2) $$\frac{\partial \theta }{\partial h}=-\frac{H}{C_{p}\cdot \rho \cdot U_{*}\cdot k}\cdot h^{-1}\;\mathrm{for}\;0<h<0.03L$$

(3) $$\frac{\partial \theta }{\partial h}=-0.027\cdot H^{\frac{2}{3}}\cdot h^{-\frac{4}{3}}\;\mathrm{for}\;0.03L<h<L$$

(4) $$\frac{\partial \theta }{\partial h}=0\;\;\;\mathrm{for}\;h>L$$

In these expressions, *h* denotes the height above ground (m), *k* is the Von Karman constant (0.4, dimensionless), *C_p_* and *ρ* are the specific heat (J/kg C) and density of air (kg/m^3^), respectively, U_*_ represents the friction velocity (m/s^−1^), and *L* is the Obukhov length (m).

The friction velocity (U_*_), which is a scaling parameter related with the vertical flux of horizontal momentum in the surface layer [30], is obtained as follows:

(5) $$U_{*}=\frac{k\cdot U}{ln(h_{0}/z_{0})}\;$$

*U* represents the wind speed (m/s), *h*_0_ is the height at which the wind speed is measured (station’s height, in m), and *z*_0_ is the surface roughness parameter (m). The value of *z*_0_ can be retrieved from tables in the literature [33].

The Obukhov length (*L*) indicates the height above which heat flux no longer affects air temperature. It can be derived from:

(6) $$L=-87\cdot 10^{3}\cdot \frac{U_{*}^{3}}{H}$$

From the potential temperature gradient (*∂θ/∂h*), the actual temperature gradient *(∂T/∂h*) can be derived using meteorological parameters with the following equation:

(7) $$\frac{\partial T}{\partial h}={\left(\frac{P}{1000}\right)}^{0.286}\cdot \left(\frac{\partial \theta }{\partial h}+2.06\cdot \frac{T}{P^{1.286}}\cdot \frac{\partial P}{\partial h}\right)$$

In this expression, *P* is the atmospheric pressure (hPa), T corresponds to the temperature (C), and *∂P/∂h* is the vertical gradient of atmospheric pressure (hPa/m).

Finally, the refractivity gradient (*∂N/∂h*), assuming that humidity and CO_2_ gradients with respect to height are negligible, can be determined from:

(8) $$\frac{\partial N}{\partial h}=\frac{\partial N}{\partial T}\cdot \frac{\partial T}{\partial h}+\frac{\partial N}{\partial P}\cdot \frac{\partial P}{\partial h}$$

The derivatives of refraction with respect to temperature (*∂N/∂T*) and pressure (*∂N/∂P*) are obtained by differentiating the corresponding expression used to calculate refractivity (see Section 3).

### 2.3. ERA-5 for Sensible Heat Flux Estimation

The sensible heat flux (H) is a critical parameter in the Turbulence Transfer Model. It represents the transfer of heat between the Earth’s surface and the atmosphere through the effects of turbulent air motion. Heat flux processes play an important role in various environmental processes and their understanding is crucial for weather forecasting and climate-related research. In our study, the significance of the sensible heat flux lies in its influence on the vertical behavior of the atmosphere and, consequently, the estimation of refractivity gradients.

Various methods exist for estimating sensible heat flux. The eddy-covariance method is one of the most established. This method is based on the covariance in the temperature and the vertical component of wind velocity [34]. The application of the Monin–Obukhov similarity theory offers another approach to derive sensible heat flux through the in situ measurement of the structure parameter of the refractive index ($C_{n}^{2}$) with instruments such as optical scintillometers based on the analysis of the fluctuations in two laser beams [35].

These techniques require specific instruments and processing strategies that lead to an increase in cost and they are often impractical in geodetic campaigns. As an alternative, the use of remote sensing products has been investigated. Several products provide sensible heat flux information, including the Japanese 55-year reanalysis dataset (JRA55) produced by the Japan Meteorological Agency, or the National Centers for Environmental Prediction reanalysis (NCEP1 and NCEP 2), and Modern-Era Retrospective Analysis for Research Applications (MERRA) [36,37].

Among these, ERA5, developed by the European Centre for Medium-Range Weather Forecasts (ECMWF) as part of the Copernicus Climate Change Service (C3S), offers a detailed record of global atmospheric, land surface, and ocean wave parameters since 1950, with a spatial resolution of 31 km and hourly data availability. Several studies have evaluated different parameters within ERA5, including sensible heat flux. Comparisons between energy fluxes from ERA5 and its predecessor, ERA-Interim (ERA-I), indicate that ERA5 provides more accurate estimates compared to direct measurements [38]. Regarding the specific variable of sensible heat flux, ERA5 offers better accuracy than the ERA5-land product [39].

Although ERA5’s spatial resolution of 31 km and temporal resolution of 1 h may seem coarse, it suits our study, which only requires an efficient estimation of overall gradients.

**Figure 1:**
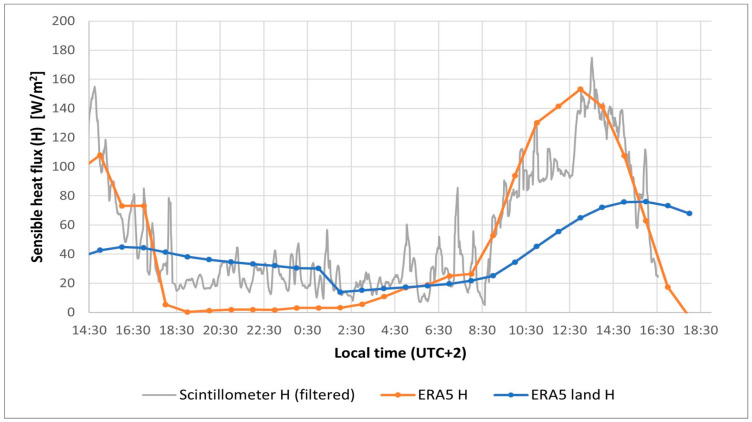
Evolution of measured sensible heat flux and that retrieved from ERA5 and ERA5-land.

For a better assessment of the quality of the retrieved values of sensible heat flux from ERA5 and ERA5-land, we compared their values with those measured using a scintillometer for approximately 28 h. The measurements were conducted over 600 m of asphalt on the 23–24 September 2023 on a former airport facility in Interlaken (Switzerland). The scintillometer used was a BLS900 from Scintec (Tübingen, Germany). The evolution of the measured sensible heat flux (after some filtering to reduce the original noise) and the ERA5 and ERA5-land values are presented in Figure 1. During the daytime, the ERA5 values closely match the measured ones. During the night, however, ERA5 tends to underestimate the sensible heat flux compared with the measurements, while the ERA5-land values are slightly higher.

Although these results are specific to the location and time of the experiment and cannot be generally extrapolated to other situations, the good agreement between ERA5 and the measured values suggests that ERA5 is a reliable source for estimating the sensible heat flux. This, along with the favorable results seen in the literature and ERA5’s easy accessibility, motivates our choice of this dataset as a tool for deriving sensible heat flux values needed for our model.

It is important to acknowledge that the validation experiment was conducted over an asphalt surface, while our field campaigns involved measurements over water. The different thermal properties of these surfaces can influence sensible heat flux behavior, potentially leading to differences in atmospheric gradients.

In the context of this study, the sensible heat flux is just one of several parameters used, along with meteorological measurements. As the final refractivity gradient results from the combined influence of these variables, small deviations in the heat flux estimation have a limited impact on the overall correction.

## 3. Three-Dimensional Refractivity Model (3D-RM) Approach

**Figure 2:**
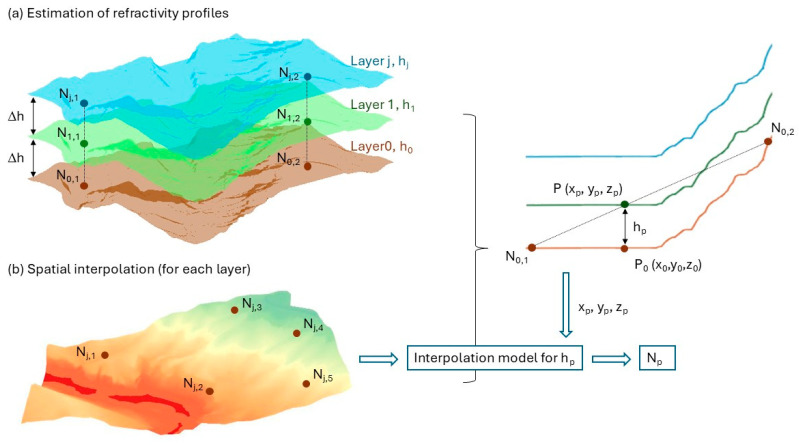
Model basis: (**a**) estimation of refractivity profiles or layering, (**b**) spatial interpolation for each layer.

Our proposed model (3D-RM) is based on two key principles: vertical layering and spatial interpolation. Firstly, the TTM is applied on each meteorological station for vertical characterization of both refractivity values and gradients. Secondly, the refractivity information obtained is used to perform spatial interpolation at specific heights. These values are subsequently integrated for each baseline measured. Figure 2 illustrates the basis of the model with these two fundamental ideas.

Meteorological sensors are deployed at specific locations within the study area, each installed at a height above ground (*h*_0,*i*_), where *i* represents each meteorological sensor. For practical purposes, the sensors are assumed to be positioned at approximately the same height, denoted as *h*_0_. However, when sensors are installed at significantly different heights above ground (i.e., with differences exceeding the chosen interval between layers, Δh), an additional adjustment is required to align the refractivity values to a common reference height *h*_0_. This adjustment is achieved using the TTM, analogous to step 1 of the model.

At each meteorological station *i*, the refractivity at *h*_0_ (denoted *N*_0,*i*_) is calculated using local meteorological parameters. Since the interaction between the Earth’s surface and the lower atmosphere decreases with height, the atmosphere is divided into layers that are approximately parallel to the Earth’s surface at discrete heights above ground level (1 m, for instance). The vertical refractivity gradient is estimated for each layer by applying the TTM, which is subsequently used to propagate the refractivity from the lower layer to the upper ones, resulting in a set of refractivity values for discrete vertical layers at each meteorological station.

For each particular layer, spatial interpolation is performed to estimate the refractivity at any point P inside the study area at this specific height so that an interpolation model is constructed for each layer.

**Figure 3:**
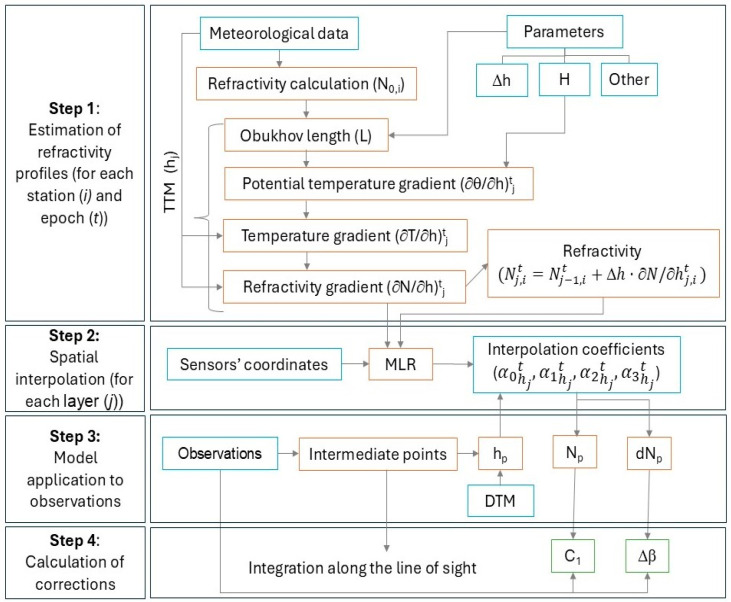
Workflow of the 3D-RM.

The model implementation is divided into four steps: estimation of refractivity profiles, spatial interpolation at each layer, model application to observations, and calculation of distance and vertical angle corrections. The overall workflow of the model is shown in Figure 3.

### 3.1. Step 1—Estimation of Refractivity Profiles

The initial step of the model involves preparing vertical refractivity profiles across multiple height layers at each meteorological station. These profiles represent the refractivity values at different heights, calculated using the TTM. The TTM requires specific input data, including the meteorological parameters (temperature, pressure, humidity) automatically collected at regular intervals, the sensor’s height above the ground (*h*_0,*i*_), the value of sensible heat flux (*H*), vertical height increments (Δ*h*), maximum height (*h_max_*), and other parameters such as the surface roughness and wind velocity.

For each meteorological station *i*, the refractivity value at *h*_0_ (*N*_0,*i*_) is computed epoch by epoch using the following equation for visible and near-infrared wavelength for EDM to within 1 ppm [5]:

(9) $$N=N_{gr}\cdot \left(\frac{273.16}{1013.25}\right)\cdot \frac{P}{T}-11.27\cdot \frac{e}{T}\;$$

where *N_gr_* is the group refractivity (dependent on the instrument wavelength, dimensionless), *e* is the partial water pressure (related to humidity, hPa), and *T* and *P* represent again the temperature (K) and pressure (hPa), respectively.

Then, the TTM is applied to calculate refractivity gradients across different atmospheric layers for each meteorological station. The layers extend from the sensor height (*h*_0_) up to the maximum specified height (*h_max_*) with values calculated at regular vertical intervals (Δ*h*). The TTM (see Section 2.2) first estimates potential temperature gradients (*∂θ/∂h*) (Equations (1)–(3)), which are then converted to temperature gradients (*∂T/∂h*) (Equation (7)). Finally, these temperature gradients are used to compute the refractivity gradients (*∂N/∂h*). The refractivity gradients are calculated by applying the refractivity formula in Equation (9) to the general formula in Equation (8). After certain simplifications, such as the assumption of a constant vertical pressure gradient of −0.12 hPa/m, the expression results in:

(10) $$\frac{\partial N}{\partial h}=\left(\left(15.02\cdot e-79.53\cdot P\right)\cdot \frac{1}{T^{2}}\right)\frac{\partial T}{\partial h}+\left(79.53\cdot \frac{1}{T}\right)\left(-0.12\right)\;$$

With the refractivity values calculated from sensor data at *h*_0_ and the refractivity gradients determined using the TTM, refractivity values at successive layers are estimated for each meteorological station. Starting from *N*_0_, the refractivity at the next layer (*N*_1_) is calculated by adding the refractivity gradient at that layer multiplied by the height interval. This process is iteratively repeated for each layer. In this way, refractivity is estimated at each desired height. This procedure is carried out for every time for which meteorological data are available. The calculated refractivity values and gradients at different heights and times are then stored, for subsequent use in the next step of the model.

### 3.2. Step 2—Spatial Interpolation at Each Layer

The second step of the model is the spatial interpolation of both the refractivity and the refractivity gradient at the designated heights or layers above ground (*h_j_*), ranging from *h*_0_ to *h_max_*, with an increment in Δ*h*.

After evaluating different interpolation methods, Multiple Linear Regression (MLR) was selected for it proved effective for accurate refractivity interpolation [40]. In this case, the dependent variable (refractivity) of the linear model is determined from a set of independent variables (3D coordinates).

The use of 3D coordinates is important to adequately capture the spatial variability in refractivity, particularly in regions with varying terrain elevation. This approach ensures that both spatial variability (including elevation) and influence of the ground (through specific models for different heights above ground) are considered. In our specific context, coordinates are expressed in a local system, being x and y planimetric coordinates, and z representing altitude (height above a reference level).

The expression for each of these models is as follows:

(11) $$N_{h_{j}}^{t}={\alpha_{0}}_{h_{j}}^{t}+{\alpha_{1}}_{h_{j}}^{t}\cdot x+{\alpha_{2}}_{h_{j}}^{t}\cdot y+{\alpha_{3}}_{h_{j}}^{t}\cdot z$$

The coefficients (${\alpha_{0}}_{h_{j}}^{t},{\alpha_{1}}_{h_{j}}^{t},{\alpha_{2}}_{h_{j}}^{t},{\alpha_{3}}_{h_{j}}^{t}$) are derived for each height level (*h_j_*) and for each time (*t*) computed in the previous step of the model. To determine the MLR model by least-squares adjustment, data from the meteorological stations are used, where each station’s coordinates and their refractivity values calculated for the specific height above ground *h_j_* are inputs.

The spatial domain for interpolation is typically bounded by the extent of the sensor network. Points within this domain are interpolated, while points outside may result in extrapolation. However, extrapolation must be avoided to minimize errors as it involves estimating values beyond the range of observed data.

For evaluation purposes, each fitted model’s root mean squared error (RMSE) and the coefficient of determination (R^2^) are computed and stored. These metrics provide insight into the model’s accuracy for each height level and time.

### 3.3. Step 3—Model Application to the Observations

Once the interpolation coefficients for each height have been obtained, the model can estimate the refractivity and its vertical gradient at any point within the working area. This initial version of the model is designed to be applied to distance and vertical angle outdoor measurements (e.g., total station measurements).

To apply the model to field observations, approximate coordinates for both the station and the target point are necessary. Typically, measurements are made from a point with known coordinates to one with an unknown position; so, the coordinates of the target point are calculated from observations (distance, vertical and horizontal angles). The station’s orientation is assumed to be known.

In order to correct these measurements, both refractivity and the vertical refractivity gradient must be evaluated along the line of sight (LoS) between the station and the target point. For distance corrections, *N* is integrated along the LoS, while vertical angle corrections rely on the refractivity gradient perpendicular to LoS, which can be expressed in terms of vertical refractivity gradient (see Section 3.4).

As a first approximation of the sight path, the straight LoS is discretized into a series of intermediate points at defined intervals (e.g., every 100 m). For each intermediate point, the following steps are implemented to estimate the refractivity and the vertical refractivity gradient:

1. The 3D coordinates (*x*,*y*,*z*) of each intermediate point are calculated (in a local Cartesian System).
2. The corresponding altitude at the (*x*,*y*) location, referred to as (*h_DTM_*), is determined using a Digital Terrain Model (DTM). The DTM provides altitude in a specific system, usually orthometric height.
3. The height above the ground of the intermediate point (*h_p_*) is calculated as the difference between the point’s *z*-coordinate and the reference *z*-coordinate, in this case, the DTM altitude of the point at ground level (see Figure 2). It is crucial to ensure consistency between the coordinate system used for the computed points’ coordinates and the DTM. If different systems are used, such as local coordinates for the points and orthometric height in the DTM, a transformation must be applied to relate them.
4. Based on the computed height above ground (*h_p_*) and the observation time, interpolation coefficients for the nearest height layer (*h_j_*) are retrieved.
5. The retrieved coefficients are applied to the intermediate point’s coordinates to estimate refractivity and its gradient at that specific location and time.

### 3.4. Step 4—Calculation of Distance and Vertical Angle Corrections

In the last step of the model, distance and vertical angle corrections are obtained for each particular observation by integrating the refractivity and refractivity gradients estimated at all the intermediate points of the LoS. Below, distance and angle corrections are furtherly explained.

The distance correction is related to the index of refraction as follows [7]:

(12) $$c_{1}=D_{0}\cdot \left(\frac{n_{0}}{n_{m}}-1\right)$$

where *D*_0_ is the measured distance, *n*_0_ is the reference index of refraction, which depends on the instrument, for example, 1.000286338 for a Leica TM30 TS [41] (Wetzlar, Germany).

When *n_m_* is the average refractive index of the baseline ends, this correction is known as the first velocity correction, and it needs to be refined with the second velocity correction in high-accuracy applications [7]. As we use estimations along the line of sight, no refinement is needed.

The mean index of refraction nm is computed by integrating the values along the entire path (l). The integral is solved numerically using the trapezoidal method:

(13) $$n_{m}=\frac{1}{l}\int_{0}^{l}n\left(s\right)\cdot s=\frac{1}{l}\;\left(\frac{∆s}{2}\left[n_{0}+2\cdot n_{1}+\cdots +2\cdot n_{k-1}+n_{k}\right]\right)$$

As the interval is a fixed value, the last step from the last intermediate point to the target point will introduce an error as it will be shorter than the defined interval. For simplicity, this error is assumed to be negligible. The refraction angle, defined as the change in the vertical angle due to refraction, is directly related to the refractivity gradient along the LoS. Since points near the station have a greater influence on the refraction angle, a weighting of the refractivity gradient is necessary.

To calculate the refraction angle (∆β) from refractivity gradients (*∂N/∂h*), we use the following equation [31]:

(14) $$∆\beta =\frac{10^{-6}}{l}\cdot \cos\beta \cdot \int_{0}^{l}\frac{\partial N}{\partial h}\cdot \left(l-s\right)\cdot ds$$

where *l* is the trajectory length, β is the vertical angle from the horizontal plane to the LoS, and *s* is the distance from the station.

In this expression, cos⁡β adjusts the refractivity gradient along the LoS to account for the typical case of non-horizontal trajectories. The curvature of the LoS is proportional to the gradient of refractivity in the perpendicular direction (*∂N/∂q*), which can be approximated as *∂N/∂q =* cos⁡β·*∂N/∂h*, leading to Equation (14).

The integral in Equation (14) is evaluated again using the trapezoidal method, with the gradient interpolated at each intermediate point. The calculated refraction angle is then added to the measured zenith angle to correct it.

## 4. Experimental Study

### 4.1. Study Site: La Muela in Cortes de Pallás (Spain)

The area selected for the assessment of the proposed 3D refractivity model (3D-RM) is La Muela in Cortes de Pallás (Spain), where the Diputació de València and the Universitat Politècnica de València have collaborated on a long-term deformation monitoring project since 2017 [42].

Among the reasons that explain why this area perfectly suits the purpose of this study, we can highlight two: first, the complex topography, which includes a cliff and a water reservoir, prevents the installation of meteorological sensors at critical points, such as those on the cliff; and second, the existence of a well-established geodetic network of ten pillars in the area, which have been monitored over several years, and may well serve as reference points. In addition to the geodetic network, 15 prisms are mounted on the rock for long-term monitoring [42]. For this study, a selection of these prisms located on a specific section of the cliff are used as control points (CPs).

The geodetic network and the 15 CPs were periodically measured using high-precision methodology with a Kern Mekometer ME5000, an instrument with sub millimetric accuracy. This process involves measuring distances and a rigorous network adjustment, resulting in highly accurate coordinates for both the geodetic pillars and the cliff reflectors. The accuracy of the coordinates for the geodetic pillars is better than 1 mm and 3 mm in the horizontal and vertical components, respectively [41], while the accuracy for the 15 CPs is slightly lower due to measurement geometry, the type of prism used, and the fact that no meteorological sensor can be installed on the cliff.

As part of previous monitoring campaigns [42,43], these reference coordinates were carefully validated and complemented by information on any potential displacements over time. In this study, they serve as “absolute” values for calculating theoretical distances and angles, providing a baseline for evaluating the accuracy of the proposed atmospheric correction models. However, it is important to consider that small displacements may have occurred since the last field campaign.

### 4.2. Instruments and Experiment Design

The 3D-RM can potentially be applied to different geodetic techniques (EDM, TS) and is not limited to well-controlled target points realized by reflectors. However, for evaluating both accuracy and precision, we decided to use a robotic total station set up over a known point to measure 10 reflectors on the cliff whose coordinates are available from previous campaigns.

Two campaigns were performed to test the model. The first campaign took place on 20 July 2023 and was focused on obtaining the initial results of the model. During this campaign, 5 automatic measurement series were taken at approximately one-hour intervals from 8 AM to 11 AM. The second campaign, carried out on 25 June 2024, was planned to cover a broader time range to better analyze the effects of refraction, from sunrise to sunset. In this campaign, 5 series were automatically recorded each hour from approximately 7 AM to 10 PM.

**Figure 4:**
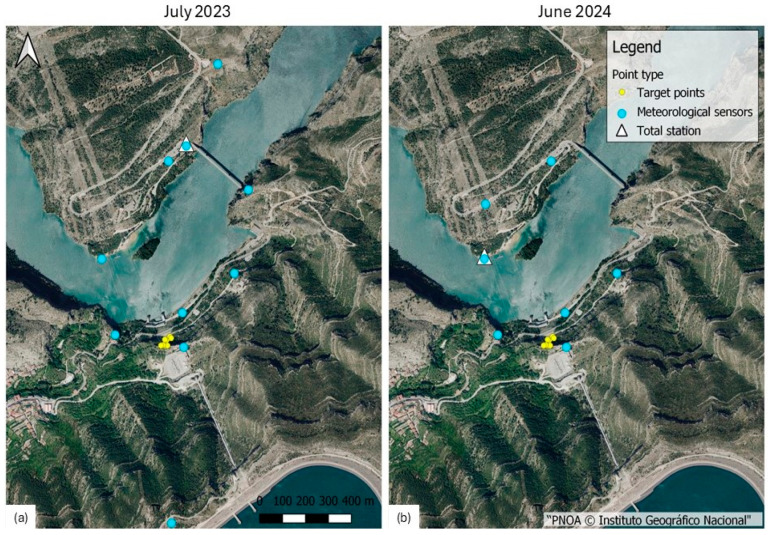
Locations of the involved points for both campaigns: (**a**) July 2023, and (**b**) June 2024.

The location of the different points involved in the two experiments is shown in Figure 4.

A Leica TM30 robotic total station was used to measure the distances to and angles of 10 of the prisms in the cliff. The total station was mounted on a tripod over a permanent benchmark (named 9000). In the June 2024 campaign, the total station was set up on one pillar of the geodetic network (point 8009). The measurement series were recorded automatically, using dual-face observations. At this point, it is worth noting that this type of experiment requires samples large enough to be considered statistically significant, which requires the use of automatic measuring. Unfortunately, as the vertical angles were collected by using an automated target recognition system (ATR), which involves internal algorithms that conceal the influence of refraction, they cannot be considered reliable raw measurements for 3D-RM validation. Moreover, current TSs cannot easily isolate vertical refraction from other potential sources of error such as limitations in the vertical compensator or vertical deflection. On the contrary, raw distances clearly show the effect of refraction and, thus, can be safely used for validation.

**Figure 5:**
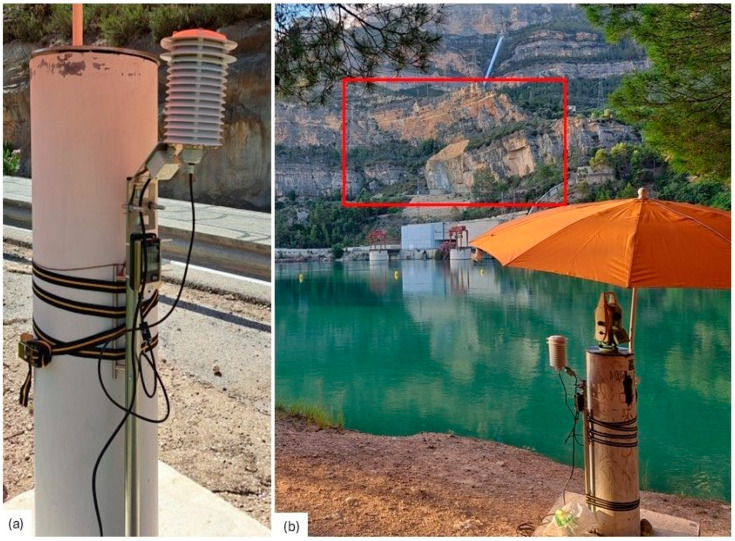
(**a**) Detail of a meteorological sensor with self-ventilated shelter. (**b**) Total station and meteorological sensor in point 8009 in the June 2024 campaign. The area of target points is marked in red.

Regarding meteorological sensors, Testo 176P1 data-loggers (Titisee-Neustadt, Germany), previously calibrated at the UPV calibration laboratory, were used. They were installed within self-ventilated shelters and firmly attached to the selected pillars of the geodetic network (Figure 5). During the July 2023 campaign, 10 data-loggers were installed, while in June 2024, only 7 were deployed. The sensors were programmed to automatically record temperature, humidity, and air pressure at 60 s intervals, which were considered suitable for efficient modeling of the local meteorological conditions.

### 4.3. 3D-RM Parameters

The present model allows for the configuration of several parameters to tailor the processing to the specific site conditions. For this study, the following parameter values were selected:

- Point interval: 100 m. For each observation, in the station–target line of sight, a point is calculated every 100 m. Theoretically, meteorological parameters should be measured every few hundred meters [20]. According to this recommendation and the characteristics of the area, a 100 m interval was deemed appropriate.
- Vertical interval: 1 m. The TTM is applied to obtain refractivity values for each meter. This value was chosen particularly focusing on the lower part of the boundary layer where the influence of the surface is strongest. For higher heights (above 20–30 m), a greater separation between layers could be used.
- Maximum height: 200 m. This value was chosen based on the terrain profiles in the area to ensure that all the points of the lines of sight remain within 200 m above ground level.
- Wind speed: given the stable wind conditions during the observation campaigns, with values ranging from 2 to 5 m/s, a constant wind speed of 3 m/s was adopted as a representative average.
- Roughness parameter: A value of 0.02 m was used to represent the terrain. This value corresponds to open terrain [33].
- Heat flux interpolation: ERA5 provides one heat flux value per hour; linear interpolation was applied to estimate the heat flux at the time of each observation.

## 5. Results and Discussion

The analysis is designed to assess model performance in terms of precision (repeatability under similar conditions), and accuracy (reproducibility under different conditions). As explained in Section 4.2, only raw distances could be used for this purpose because the internal preprocessing of vertical angle measurements with algorithms that are not publicly accessible conceals the influence of refraction. To assess the model precision, the standard deviations are analyzed, while proximity to reference values is used to evaluate its accuracy. The model’s performance is also compared against the standard correction method, which is referred to as “St” (from station) throughout this section. Similarly to many deformation monitoring projects with non-accessible target points, in this case, the standard method only uses meteorological data collected at the station.

Since this study also seeks to determine the potential and possible limitations of the model, two versions of the model were tested: the first version (3D-RM) incorporates meteorological data from all the installed sensors, including the station point; the second version (3D-RM2) excludes the station sensor, estimating its value from the remaining sensor network. If this second version proved valid, the model could be efficiently used with a limited number of sensors. Moreover, they could be statically located in those points that better characterize the local atmosphere instead of using measuring stations which may not be optimally distributed for refraction mitigation.

**Figure 6:**
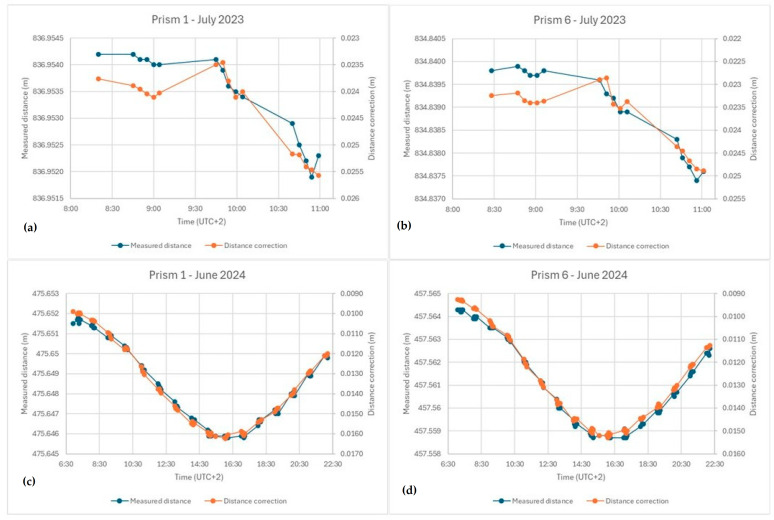
Evolution of the measured distances and the corresponding corrections: (**a**) point 1, July 2023; (**b**) point 6, July 2023; (**c**) point 1, June 2024; (**d**) point 6, June 2024.

Figure 6 shows the variation in measured distances and the correction obtained over time for target points 1 and 6 during both campaigns (July 2023 and June 2024) using the 3D-RM. Please note the decreasing values in the distance correction axis.

The corrections obtained from the model closely align with the variations in the measured distances, which are affected by refraction, so that very stable values are obtained for the raw distances plus the corrections. In the experiment conducted in July 2023 (graphs a and b), it can be observed that variations in measured distance and corrections generally correspond to around 0.5 mm. Even small changes or tendencies in distances, such as those around 9 AM, are reflected in the model’s corrections.

For the field campaign carried out in June 2024 (graphs c and d), covering almost 16 h, the evolution of the refraction effects is more pronounced, amounting to 6 mm, but the correspondence between distance variations and corrections remains within 0.5 mm for the entire period.

Table 1 summarizes the distance corrections obtained for both campaigns using the standard approach and the two versions of the model, along with the reference distances.

As expected, corrections for the July 2023 campaign are larger than those obtained for the June 2024 campaign due to the longer distances measured, which were around 800 m and 450 m, respectively. Overall, the 3D-RM models yield larger corrections than the standard method. Interestingly, differences between 3D-RM and 3D-RM 2 are negligible, which shows that the refraction model is reliable enough to even eliminate the need for a meteorological station set up next to the total station.

The standard deviation results for measured distances and distances corrected by each method are presented in Table 2.

It is important to note that the standard deviation is a reliable measure when there is a sufficiently large dataset. In our study, the July 2023 campaign contained only a few series, while in June 2024, the time range covered all the daytime. All correction methods lead to significant improvements over raw field measurements, with standard deviations consistently reduced.

To evaluate the results in absolute terms, the known coordinates of the points from previous campaigns, together with the deformation analysis, were used to calculate theoretical distances. Table 3 shows the differences between the distances (raw and corrected) and the reference values.

The results clearly show the necessity of applying atmospheric corrections since the raw distances taken in the field without correction (Dfield) are tens of millimeters away from their true values. For the July 2023 campaign, 3D-RM improves the accuracy by approximately 2 mm as compared to the standard approach (St), demonstrating the model’s effectiveness over longer baselines (around 800 m).

In the June 2024 campaign, where distances were shorter (around 450 m), the results were less consistent, with smaller improvements over the standard approach. This is likely because, for shorter baselines, the impact of atmospheric refraction is reduced and the traditional approach—based on meteorological data from the station—becomes closer to reality. Despite this, the evolution of the refraction correction throughout the day aligns well with the expected atmospheric behavior, indicating that the 3D-RM captures the temporal variability in atmospheric conditions effectively.

Finally, there are no substantial differences between the 3D-RM and 3D-RM 2 models, suggesting that a well-distributed network of meteorological sensors can sufficiently support the model without requiring a sensor at the station point.

## 6. Conclusions and Future Work

The traditional approach to mitigate the effect of atmospheric refraction, based only on meteorological parameters collected at the end points of the measurement line, has proven to be not optimal for long-range geodetic measurements, especially in critical applications like deformation monitoring with complex areas where the target points are typically inaccessible.

This paper introduces the 3D refractivity model (3D-RM), which is based on current technology and easily available data products, to estimate refractivity and refractivity gradients at every point within a working area covered by a network of meteorological sensors. The model operates by dividing the line of sight (LoS) into intermediate points where refractivity and refractivity gradients are interpolated using vertical layering from the Turbulence Transfer Model (TTM) and spatial interpolation within each layer, performed via Multiple Linear Regression (MLR). These interpolated values can be then integrated to obtain automatic corrections for both distance and vertical angle measurements.

As shown in the application, the 3D-RM performed well in the field campaigns, providing reliable corrections even when no meteorological data were available at the target points. The second version of the model (3D-RM 2), which estimates corrections without using the sensor at the measuring station, produced nearly identical results to the full-sensor-based model. This feature makes the model highly adaptable even though only a limited number of meteorological sensors are available. In addition, the sensors can be better located in those points that better characterize the local refraction, which do not necessarily coincide with the measuring stations. By reducing the number of required sensors to cover just the area of interest, the model enables rapid and cost-effective measurements without the need to relocate sensors, significantly reducing setup time and operational costs.

For distance measurements, the refraction corrections obtained in the first campaign (with distances around 800 m) are in the order of 2 cm, while the second campaign (with distances around 450 m) yielded corrections in the order of 1 cm. These corrections from the 3D-RM were generally slightly higher than those from the standard approach by using meteorological data only at the station point. In terms of standard deviation, while the improvements relative to the standard method were not always significant, the values obtained still met the precision of the instrument.

As regards accuracy, defined as closeness to reference values, a general improvement of around 2 mm was observed in the July 2023 campaign when using the 3D-RM compared to the standard approach. In the June 2024 campaign, the results proved less significative, with improvements at only some target points. Even so, the differences compared to the standard approach were not significant. Additionally, 3D-RM2 delivered almost identical results to the original 3D-RM model, which reinforces the practicality and effectiveness of the method.

The use of ERA5 data for estimating sensible heat flux proved to be a valuable component in the vertical layering of the model. This integration allowed for accurate vertical refractivity profiles within the boundary layer without increasing the costs of the processing and the field campaigns. However, some limitations of ERA5 must be acknowledged, particularly its spatial resolution of 31 km and the possible inaccuracies when applied to measurements over water surfaces, where thermal properties differ significantly from those of land. While ERA5 serves as a practical first estimation of heat flux, future work should include a more detailed assessment of its performance over water, as well as comparisons with other datasets and direct measurement techniques. Expanding this analysis will help refine the model’s accuracy and adaptability to varying surface and atmospheric conditions.

Additionally, while the model’s performance is promising, there is room for future work, such as additional testing in a broader range of atmospheric conditions in order to better understand the model’s robustness and limitations. Validation over longer distances in a long time period would be especially valuable as longer baselines tend to amplify atmospheric effects and provide clearer insights into the model’s performance.

Further, due to the use of automatic ATR systems in our field experiments, we were unable to evaluate the model’s performance on vertical angle corrections as the recorded angles were automatically adjusted by the instrument’s internal algorithms. For future evaluations, vertical angle measurements should be conducted without ATR, taking manual measurements instead to ensure the angles reflect the actual refractive effects.

In conclusion, the 3D-RM offers a flexible, cost-effective approach to atmospheric refraction mitigation, demonstrating strong potential for enhancing the accuracy of geodetic measurements. By addressing the identified limitations through future studies, the model’s applicability and reliability can be further strengthened, making it a valuable tool for a wide range of geodetic applications.

## Figures and Tables

**Table 1:** Reference distances and corrections obtained.

Point	July 2023				June 2024			
	Dref [m]	Distance Corrections [mm]			Dref [m]	Distance Corrections [mm]		
		St	3D-RM	3D-RM2		St	3D-RM	3D-RM2
1	837.0030	22.2	24.3	24.3	475.9862	11.9	13.6	13.8
2	835.7635	22.2	24.2	24.2	477.3272	11.9	13.6	13.8
3	835.2299	22.2	24.3	24.3	482.8800	12.0	13.8	14.0
4	834.8463	22.2	23.8	23.8	462.3186	11.5	12.9	13.1
5	831.9391	22.1	23.6	23.6	461.6867	11.5	12.8	13.1
6	834.8872	22.2	23.7	23.7	457.5971	11.4	12.7	12.9
7	807.9628	21.5	22.5	22.5	449.8426	11.2	12.2	12.5
8	791.8359	21.0	21.8	21.8	446.7363	11.1	12.0	12.2
9	798.4321	21.2	22.2	22.2	457.1084	11.4	12.4	12.6
15	806.4697	21.4	22.4	23.3	442.8837	11.1	12.0	12.2

**Table 2:** Distance standard deviations.

Point	July 2023				June 2024			
	Standard Deviation [mm]				Standard Deviation [mm]			
	D_field_	St	3D-RM	3D-RM 2	D_field_	St	3D-RM	3D-RM 2
1	0.8	0.5	0.3	0.3	2.5	0.2	0.2	0.3
2	0.8	0.5	0.3	0.3	1.6	0.1	0.2	0.3
3	0.9	0.5	0.4	0.4	1.3	0.3	0.1	0.3
4	0.9	0.5	0.4	0.4	2.2	0.3	0.2	0.3
5	0.9	0.5	0.4	0.4	2.0	0.2	0.2	0.3
6	0.9	0.5	0.4	0.4	1.9	0.2	0.2	0.3
7	0.9	0.4	0.4	0.4	2.0	0.2	0.2	0.2
8	0.8	0.5	0.4	0.4	1.9	0.2	0.2	0.2
9	0.8	0.5	0.4	0.4	1.9	0.2	0.2	0.3
15	1.1	0.3	0.5	0.5	1.9	0.2	0.2	0.2

**Table 3:** Differences between distances (measured and corrected) compared to reference values.

Point	July 2023				June 2024			
	Differences with Reference Values [mm]				Differences with Reference Values [mm]			
	D_field_	St	3D-RM	3D-RM 2	D_field_	St	3D-RM	3D-RM 2
1	−26.5	−4.2	−2.1	−2.1	−14.8	−3.7	−2.0	−1.7
2	−25.4	−3.2	−1.2	−1.2	−11.4	−0.3	1.4	1.7
3	−27.4	−5.2	−3.1	−3.1	−9.9	1.5	3.2	3.5
4	−24.7	−2.6	−1.0	−0.9	−13.0	−2.2	−0.8	−0.5
5	−25.5	−3.4	−1.9	−1.9	−12.7	−1.9	−0.6	−0.3
6	−25.1	−2.9	−1.5	−1.4	−13.0	−2.3	−1.1	−0.8
7	−24.3	−2.8	−1.9	−1.8	−9.1	1.4	2.4	2.6
8	−24.5	−3.5	−2.8	−2.8	−10.5	0.0	0.8	1.0
9	−21.7	−0.5	0.5	0.4	−8.3	2.5	3.4	3.7
15	−21.7	−0.3	0.6	0.7	−7.4	3.0	3.8	4.1

## Data Availability

The hourly ERA5 reanalysis data used in this study are provided by the Copernicus Climate Data Sore and can be downloaded from https://cds.climate.copernicus.eu/datasets/reanalysis-era5-single-levels?tab=overview. The DTM used in this study is provided by the Instituto Geográfico Nacional (IGN) and is available at https://centrodedescargas.cnig.es/CentroDescargas/index.jsp. The raw data that support the findings of this study are openly available at the following URL/DOI: https://zenodo.org/records/14501909.

## References

[1] Ingensand H. Concepts and Solutions to Overcome the Refraction Problem in Terrestrial Precision Measurement. Proceedings of the FIG XXII International Congress.

[2] Ciddor P.E. (1996). Refractive Index of Air: New Equations for the Visible and near Infrared. Appl. Opt..

[3] Ciddor P.E. (2002). Refractive Index of Air: 3 The Roles of CO_2_, H_2_O, and Refractivity Virials: Erratum. Appl. Opt..

[4] Ciddor P.E., Hill R.J. (1999). Refractive Index of Air. 2. Group Index. Appl. Opt..

[5] The International Association of Geodesy (1999). IAG Resolutions Adopted at the XXIIth General Assembly in Birmingham.

[6] Neyezhmakov P., Prokopov A. On the Accuracy of Determining the Mean Integral Refractive Index of Air by Its Values at the End Points of the Trace. Proceedings of the XXXII International Scientific Symposium Metrology and Metrology Assurance, MMA 2022.

[7] Rüeger J.M. (1992). Electronic Distance Measurement: An Introduction.

[8] Guillory J., Truong D., Wallerand J., Alexandre C. (2024). A Sub-Millimetre Two-Wavelength EDM That Compensates the Air Refractive Index: Uncertainty and Measurements up to 5 Km. Meas. Sci. Technol..

[9] Ray P., Salido-Monzú D., Wieser A. (2023). High-Precision Intermode Beating Electro-Optic Distance Measurement for Mitigation of Atmospheric Delays. J. Appl. Geod..

[10] Baselga S., García-Asenjo L., Garrigues P., Luján R. (2022). GBDM+: An Improved Methodology for a GNSS-Based Distance Meter. Meas. Sci. Technol..

[11] García-Asenjo L., Baselga S., Atkins C., Garrigues P. (2021). Development of a Submillimetric GNSS-Based Distance Meter for Length Metrology. Sensors.

[12] Brunner F.K. (1979). Vertical Refraction Angle Derived from the Variance of the Angle-of-Arrival Fluctuations. Symp.-Int. Astron. Union.

[13] Obukhov A.M. (1949). Structure of the Temperature Field in Turbulent Flow. Izv. Akad. Nauk SSSR Ser. Geogr. Geofiz..

[14] Priestley C.H.B. (1959). Turbulent Tranfer in the Lower Atmosphere.

[15] Brunner F.K., Fricke W., Teleki G. (1982). Atmospheric Turbulence and Its Effects on Direction Measurements. Sun and Planetary System.

[16] Brunner F.K. (1982). The Effects of Atmospheric Turbulence on Telescopic Observations. Bull. Géodésique.

[17] Brunner F.K. (1984). Geodetic Refraction: Effects of Electromagnetic Wave Propagation Through the Atmosphere.

[18] Friedli E., Presl R., Wieser A. Influence of atmospheric refraction on terrestrial laser scanning at long range. Proceedings of the 4th Joint International Symposium on Deformation Monitoring (JISDM).

[19] Kerekes G.A. (2023). An Elementary Error Model for Terrestrial Laser Scanning. Ph.D. Thesis.

[20] Artese S., Perrelli M. (2018). Monitoring a Landslide with High Accuracy by Total Station: A DTM-Based Model to Correct for the Atmospheric Effects. Geosciences.

[21] Wiggers D., Faggion P.L., Cruz W., Jerke A., Samir S.O. (2025). Refractive Index Analysis in Dam Monitoring: Case Study Hydroelectric power pland Governor Jayme Canet Junior. Rev. Geocienc. Nordeste.

[22] Nikolitsas K., Lambrou E. (2021). Comparison of Two Different Methodologies for Correcting Refraction in Vertical Angles. Appl. Geomat..

[23] Dodson A.H., Zaher M. (1985). Refraction Effects on Vertical Angle Measurements. Surv. Rev..

[24] Sabzali M., Jazirian I. (2022). Improvement the Modelling of Atmospheric Effects for Electronic Distance Measurement (EDM): Analysis of Air Temperature, Atmospheric Pressure and Relative Humidity of Air. Geod. Cartogr..

[25] Rodriguez F.A.C., Veiga L.A.K., Soares W.A. (2021). Temperature Acquisition System for Real Time Application of First Velocity Correction by EDM (Electronic Distance Measurement). J. Geomat. Plan..

[26] Redovniković L., Ališić I., Luketic A. (2013). Influence of Lateral Refraction on Measured Horizontal Directions. Surv. Rev..

[27] Gaifillia D., Pagounis V., Tsakiri M., Zacharis V. (2016). Empirical Modelling of Refraction Error in Trigonometric Heighting Using Meteorological Parameters. J. Geosci. Geomat..

[28] Baselga S., García-Asenjo L., Garrigues P. (2014). Practical Formulas for the Refraction Coefficient. J. Surv. Eng..

[29] Hirt C., Guillaume S., Wisbar A., Bürki B., Sternberg H. (2010). Monitoring of the Refraction Coefficient in the Lower Atmosphere Using a Controlled Setup of Simultaneous Reciprocal Vertical Angle Measurements. J. Geophys. Res. Atmos..

[30] Jordan W., Eggert O., Kneissl M. (1956). Wissenschaftliche Probleme der Trigonometrischen Höhenmessung, Handbuch der Vermessungskunde.

[31] Angus-Leppan P.V. (1979). Use of Meteorological Measurements for Computing Refractional Effects—A Review. Symp.-Int. Astron. Union.

[32] Jacobson M.Z. (2005). Fundamentals of Atmospheric Modeling.

[33] Holmes J.D. (2001). Wind Loading of Structures.

[34] Wollschläger N., Schlink U., Raabe A. (2021). A Feasibility Study for Determining the Sensible Heat Flux to and from Small Green Roofs. Boundary-Layer Meteorol..

[35] Weiss A.I., Hennes M., Rotach M.W. (2001). Derivation of Refractive Index and Temperature Gradients from Optical Scintillometry to Correct Atmospherically Induced Errors for Highly Precise Geodetic Measurements. Surv. Geophys..

[36] Chen L., Pryor S.C., Wang H., Zhang R. (2019). Distribution and Variation of the Surface Sensible Heat Flux over the Central and Eastern Tibetan Plateau: Comparison of Station Observations and Multireanalysis Products. J. Geophys. Res. Atmos..

[37] Pokhrel S., Dutta U., Rahaman H., Chaudhari H., Hazra A., Saha S.K., Veeranjaneyulu C. (2020). Evaluation of Different Heat Flux Products over the Tropical Indian Ocean. Earth Sp. Sci..

[38] Martens B., Schumacher D.L., Wouters H., Muñoz-Sabater J., Verhoest N.E.C., Miralles D.G. (2020). Evaluating the Land-Surface Energy Partitioning in ERA5. Geosci. Model Dev..

[39] Muñoz-Sabater J., Dutra E., Agustí-Panareda A., Albergel C., Arduini G., Balsamo G., Boussetta S., Choulga M., Harrigan S., Hersbach H. (2021). ERA5-Land: A State-of-the-Art Global Reanalysis Dataset for Land Applications. Earth Syst. Sci. Data.

[40] Luján R., García-Asenjo L., Baselga S. (2023). Evaluation of Interpolation Methods for Refractivity Mitigation. Environ. Sci. Proc..

[41] Leica TS30/TM30 User Manual 3.0. https://www.engineeringsurveyor.com/software/Leica_TS30_TM30_UM_en.pdf.

[42] García-Asenjo L., Martínez L., Baselga S., Garrigues P. Establishment of a Multi-Purpose 3D Geodetic Reference Frame for Deformation Monitoring in Cortes de Pallás (Spain). Proceedings of the 4th Joint International Symposium on Deformation Monitoring (JISDM).

[43] García-Asenjo L., Martínez L., Baselga S., Garrigues P., Luján R. (2023). Design, Establishment, Analysis, and Quality Control of a High-Precision Reference Frame in Cortes de Pallás (Spain). Appl. Geomat..

